# Supplementary material for: Tracking down the White Plague: The skeletal evidence of tuberculous meningitis in the Robert J. Terry Anatomical Skeletal Collection
Source: PLoS One. 2020 Mar 18;15(3):e0230418. doi: 10.1371/journal.pone.0230418 (PMC7080279; doi:10.1371/journal.pone.0230418)
Supplement: S1 Table — (MR = morgue record; DC1 = death certificate primary; DC2 = death certificate secondary; DC3 = death certificate tertiary; c. = circa; F = female; M = male; + = exhibiting GIs;– = not exhibiting GIs). (PDF) [file pone.0230418.s001.pdf]

S1 Table: Basic biographic data of individuals in the TB group (N=234).
(MR = morgue record; DC1 = death certificate primary; DC2 = death certificate secondary; DC3 = death certificate tertiary; c. = circa; F = female; M = male; + = exhibiting GIs; – = not exhibiting GIs)

No.	Terry No.	Age at death	Sex	Cause of death				Exhibiting GIs or not
				MR	DC1	DC2	DC3	
1	13R	28 years	M	Pulmonary TB	–	Infection of the right toe	–	–
2	23R	49 years	F	Pulmonary TB	–	–	–	–
3	30R	26 years	M	TB	–	TB meningitis	–	+
4	35R	60 years	M	Pulmonary TB	–	–	–	–
5	39	22 years	F	TB, pneumonia	Pneumonia	–	–	–
6	54	36 years	M	TB, diarrhoea	Pulmonary TB	–	–	–
7	84	c. 24 years	M	Pulmonary TB	Pulmonary TB	–	–	+
8	87R	73 years	F	Lues	Lues	Suspected TB	–	+
9	89R	c. 32 years	M	TB	–	–	–	–
10	90	18 years	M	–	Ichthyosis	Pulmonary TB	–	–
11	91R	c. 65 years	F	Pulmonary condition	Pulmonary TB	–	–	–
12	95	c. 35 years	F	Pulmonary TB	–	–	–	+

No.	Terry No.	Age at death	Sex	Cause of death				Exhibiting GIs or not
				MR	DC1	DC2	DC3	
13	95R	c. 30 years	M	Pulmonary TB	–	–	–	–
14	103R	c. 74 years	F	–	Emphysema	TB	–	–
15	114	43 years	F	TB	–	–	–	–
16	128	21 years	M	TB	Pulmonary TB	–	–	–
17	129	18 years	M	TB spondylitis	–	–	–	+
18	130	58 years	M	Pellagra	Pulmonary TB	–	Inanition	–
19	138	49 years	M	Pulmonary phthisis	Pulmonary TB	–	–	+
20	139	33 years	F	TB	Pulmonary TB	–	–	+
21	145R	31 years	M	Pulmonary TB	–	–	–	+
22	146R	30 years	F	Pulmonary TB	Pulmonary TB	–	Nephritis	–
23	158R	c. 32 years	F	TB	Pulmonary TB	–	–	+
24	182	43 years	M	Pulmonary TB	Pulmonary TB	–	–	+
25	194	73 years	M	Pulmonary TB	–	–	–	–
26	204	24 years	M	Pulmonary TB	–	–	–	–

No.	Terry No.	Age at death	Sex	Cause of death				Exhibiting GIs or not
				MR	DC1	DC2	DC3	
27	205	42 years	M	Pulmonary TB	Pulmonary TB	–	–	+
28	207	45 years	M	Pulmonary TB	–	–	–	–
29	220	c. 56 years	M	Pulmonary TB	–	–	–	+
30	222	20 years	M	Pulmonary TB	Pulmonary TB	–	–	+
31	230	37 years	M	Pulmonary TB	–	–	–	+
32	232R	c. 35 years	F	Pulmonary TB	–	–	–	+
33	235	29 years	M	–	Miliary TB	–	–	–
34	248R	70 years	F	Possible TB	Pneumonia	–	–	–
35	250	52 years	M	Laparotomy, stomach carcinoma	TB peritonitis	–	–	–
36	251	34 years	M	TB	Pulmonary TB	–	–	–
37	254	21 years	M	Pulmonary TB	–	–	–	–
38	255	22 years	F	Pulmonary TB	–	–	–	+
39	264	c. 67 years	M	TB, pneumonia	–	–	–	–
40	265	32 years	M	TB	–	–	–	–

No.	Terry No.	Age at death	Sex	Cause of death				Exhibiting GIs or not
				MR	DC1	DC2	DC3	
41	267	c. 42 years	M	Pulmonary TB	–	–	–	–
42	269	20 years	M	TB	–	–	–	+
43	270	55 years	M	TB	–	–	–	+
44	279	34 years	M	Pulmonary TB	–	–	–	+
45	280	24 years	F	TB	–	–	–	+
46	282	57 years	M	Myocarditis	Pulmonary TB	Myocarditis	–	–
47	283R	40 years	M	TB, haemorrhage	Pulmonary TB	–	–	–
48	284	c. 67 years	M	TB	–	–	–	–
49	304	20 years	F	–	Pulmonary TB	–	–	–
50	306	18 years	F	Pulmonary TB	–	–	–	–
51	309	45 years	M	Pulmonary TB	Pulmonary TB	TB spondylitis	Mastoiditis, sinusitis	–
52	318	45 years	M	Pulmonary TB, myocarditis	Pulmonary TB	–	–	–
53	328R	65 years	F	Possible TB, pleurisy	Pulmonary carcinoma	Undetermined	–	–
54	329	18 years	M	Pulmonary TB, syphilis	Pulmonary TB	Syphilis	–	+

No.	Terry No.	Age at death	Sex	Cause of death				Exhibiting GIs or not
				MR	DC1	DC2	DC3	
55	341	38 years	F	Pulmonary TB	–	–	–	+
56	353	c. 37 years	M	–	TB peritonitis	–	–	–
57	358R	c. 36 years	M	Pulmonary TB	–	–	–	–
58	382R	26 years	M	Pulmonary TB	–	–	–	–
59	385	21 years	M	–	Pulmonary TB	–	–	–
60	386R	66 years	M	Pulmonary TB	–	–	–	–
61	400	c. 44 years	M	TB	–	–	–	–
62	402	c. 50 years	M	TB	–	–	–	–
63	410R	35 years	M	–	Pulmonary TB	–	–	–
64	423	c. 24 years	M	TB, pericarditis	–	–	–	–
65	424	c. 30 years	M	TB, silicosis	–	–	–	–
66	426R	70 years	M	Cardiorenal disease	Arteriosclerosis	Pulmonary TB	*Diabetes mellitus*	–
67	432	67 years	M	Destructive nasal lesion	TB abscess	Pulmonary TB	–	+
68	444	c. 26 years	M	TB	Pulmonary TB	–	–	+

No.	Terry No.	Age at death	Sex	Cause of death				Exhibiting GIs or not
				MR	DC1	DC2	DC3	
69	466	31 years	M	Pulmonary TB	–	–	–	–
70	468	23 years	M	Pulmonary TB, TB spondylitis	–	–	–	–
71	490	c. 38 years	M	TB	–	Hepatic cirrhosis	–	–
72	504	c. 41 years	M	TB, bronchitis	Pulmonary TB	–	–	+
73	522	30 years	M	Pulmonary TB	–	–	–	+
74	523	47 years	M	Pulmonary TB	–	–	–	–
75	537	c. 48 years	M	Possible malaria	TB meningitis	–	–	–
76	541	28 years	F	Pulmonary TB	–	–	–	+
77	549	33 years	M	Myocarditis	Pulmonary TB	–	–	–
78	555	56 years	M	Pulmonary TB	–	–	–	–
79	562	17 years	F	Pulmonary TB	–	–	–	+
80	565	c. 32 years	M	Pulmonary TB	–	–	–	–
81	566	40 years	M	Empyema	Empyema	Possible TB	–	+
82	568	28 years	F	Pulmonary TB	–	–	–	+

No.	Terry No.	Age at death	Sex	Cause of death				Exhibiting GIs or not
				MR	DC1	DC2	DC3	
83	571	31 years	M	TB	–	–	–	–
84	572	60 years	M	Pulmonary TB	–	–	–	–
85	575	c. 47 years	M	Pulmonary TB	–	–	–	+
86	583	24 years	F	Pulmonary TB	–	–	–	–
87	585	64 years	M	Pulmonary TB	–	–	–	–
88	592	25 years	M	Pulmonary TB	–	–	–	–
89	595	25 years	M	Pulmonary TB	–	–	–	+
90	620	c. 55 years	M	Pulmonary TB	–	Myocarditis	–	+
91	621R	29 years	M	Pulmonary TB	Pulmonary TB	TB emphysema	–	–
92	626R	c. 56 years	F	Pulmonary TB	–	–	–	–
93	664	c. 48 years	M	Pulmonary TB	Pulmonary TB	Pneumonia	–	+
94	669R	72 years	M	Heart disease	Pulmonary TB	Arteriosclerosis	Heart disease	–
95	679	33 years	F	TB	–	–	–	–
96	680	30 years	F	Pulmonary TB	Pulmonary TB	TB peritonitis	–	+

No.	Terry No.	Age at death	Sex	Cause of death				Exhibiting GIs or not
				MR	DC1	DC2	DC3	
97	728R	76 years	F	Pulmonary TB, senility	–	–	–	–
98	739	40 years	M	TB, *diabetes mellitus*	Pulmonary TB	*Diabetes mellitus*	–	+
99	752	71 years	M	TB, pneumonia	Pneumoconiosis	Myocarditis	Arteriosclerosis	+
100	757	59 years	M	Pulmonary TB	–	–	–	–
101	761	c. 81 years	F	Pulmonary TB	Pulmonary TB	Myocarditis	–	–
102	771	c. 49 years	M	Pulmonary TB	–	–	–	–
103	776	c. 40 years	M	Pulmonary TB	–	–	–	–
104	786	65 years	M	Pulmonary TB	–	–	–	–
105	799	38 years	M	TB	–	–	–	–
106	820R	52 years	M	Pulmonary TB	–	–	–	–
107	822	16 years	F	Pulmonary TB	–	–	–	+
108	828	46 years	M	TB	–	–	–	+
109	844	26 years	F	Pulmonary TB	–	–	–	–
110	846	48 years	M	Pulmonary TB	–	–	–	–

No.	Terry No.	Age at death	Sex	Cause of death				Exhibiting GIs or not
				MR	DC1	DC2	DC3	
111	876	c. 22 years	M	Pulmonary TB	–	–	–	+
112	892	c. 59 years	M	Heart disease	Emphysema	Pulmonary TB	–	–
113	895	47 years	M	TB	–	–	–	–
114	896RR	26 years	F	Pulmonary TB	–	–	–	+
115	897	43 years	M	Pulmonary TB	Pulmonary TB	Myocarditis	–	–
116	902	c. 36 years	M	Pulmonary TB	–	–	–	–
117	907	41 years	F	Pulmonary TB	–	–	–	–
118	914	30 years	M	TB	–	–	–	+
119	915	27 years	M	Pulmonary TB	–	–	–	–
120	932	27 years	M	Pulmonary TB	–	–	–	–
121	933R	40 years	M	Probable TB, ascites	TB peritonitis	–	–	+
122	936	56 years	M	Pulmonary TB	–	–	–	–
123	950	59 years	M	Pulmonary TB	–	–	–	–
124	952	41 years	F	TB	–	–	–	+

No.	Terry No.	Age at death	Sex	Cause of death				Exhibiting GIs or not
				MR	DC1	DC2	DC3	
125	955	26 years	M	Pulmonary TB	–	–	–	–
126	957	56 years	F	Chest contusion	Pulmonary TB	–	–	–
127	975	60 years	M	TB, cardiac decompensation	Myocarditis	–	–	+
128	987	23 years	M	Pulmonary TB	–	–	–	+
129	1002	50 years	M	Pulmonary TB	–	–	–	–
130	1005	52 years	M	Pulmonary TB	–	–	–	–
131	1013	c. 30 years	M	Pulmonary TB	–	–	–	–
132	1018	30 years	M	Pulmonary TB	–	–	–	+
133	1020	24 years	M	Pulmonary TB	–	–	–	+
134	1027	c. 41 years	F	Pulmonary TB	–	–	–	+
135	1030	62 years	M	–	Pulmonary TB	–	–	–
136	1031	27 years	M	Pneumonia	Pulmonary TB	–	–	–
137	1033	26 years	M	Pulmonary TB	–	–	–	–
138	1034	48 years	F	Pulmonary TB	TB nephritis	–	–	–

No.	Terry No.	Age at death	Sex	Cause of death				Exhibiting GIs or not
				MR	DC1	DC2	DC3	
139	1036	38 years	M	Asthma, heart disease	Pulmonary TB	–	–	–
140	1043	46 years	M	Pulmonary TB	–	–	–	–
141	1047	c. 61 years	M	TB	–	–	–	–
142	1048	39 years	M	TB	–	–	–	–
143	1057	35 years	M	Pulmonary TB	–	–	–	+
144	1072	56 years	M	Pulmonary TB	–	–	–	–
145	1076	25 years	F	Pulmonary TB	Pulmonary TB	Pulmonary oedema	–	+
146	1086	45 years	M	Pulmonary TB	–	–	–	–
147	1093	29 years	M	Pulmonary TB	–	–	–	–
148	1095	61 years	M	Pulmonary TB	–	–	–	–
149	1096R	40 years	M	Pulmonary TB	–	–	–	+
150	1105	22 years	F	Pulmonary TB	–	–	–	–
151	1106	28 years	M	TB spondylitis, heart disease	TB spondylitis	–	–	+
152	1113	29 years	M	Pulmonary TB	Myocarditis	–	–	–

No.	Terry No.	Age at death	Sex	Cause of death				Exhibiting GIs or not
				MR	DC1	DC2	DC3	
153	1122	29 years	F	Pulmonary TB	–	–	–	–
154	1124R	49 years	F	Pulmonary TB	–	–	–	–
155	1129	50 years	F	Pulmonary TB	–	–	–	–
156	1132	67 years	M	Pulmonary TB	–	–	–	–
157	1147R	32 years	M	Pleural expansion	Possible TB, pleural effusion	–	–	–
158	1156	c. 56 years	M	Pulmonary TB	–	–	–	–
159	1157	40 years	M	Pulmonary TB	–	–	–	–
160	1159	26 years	M	Pulmonary TB	–	–	–	+
161	1165	c. 26 years	M	Pulmonary TB	–	–	–	–
162	1169	28 years	M	Pulmonary TB	–	–	–	–
163	1173	38 years	F	Pulmonary TB	–	–	–	–
164	1183	c. 21 years	M	Pneumonia	Pulmonary TB	Pleurisy	–	–
165	1185	33 years	M	Pulmonary TB	Pulmonary haemorrhage	Pulmonary TB	–	–
166	1187	21 years	M	Pulmonary TB	–	–	–	–

No.	Terry No.	Age at death	Sex	Cause of death				Exhibiting GIs or not
				MR	DC1	DC2	DC3	
167	1190	c. 64 years	M	Pulmonary TB	–	–	–	–
168	1205	38 years	M	Pulmonary TB	–	–	–	–
169	1210	46 years	F	TB	Pulmonary TB	–	–	–
170	1215	25 years	F	Pulmonary TB	Pulmonary TB	Child birth	–	–
171	1222	28 years	F	Pulmonary TB	–	–	–	+
172	1226	c. 48 years	M	Pulmonary TB	–	–	–	–
173	1230	53 years	M	Cysto-urethro-rectocele with ascites	Pulmonary TB	Pneumothorax	Cirrhosis, myocarditis	–
174	1236	36 years	F	TB	–	–	–	–
175	1247	53 years	M	TB	–	–	–	–
176	1249R	40 years	F	–	Pulmonary TB	–	–	+
177	1255	39 years	M	TB sinusitis	TB of the hips	*Delirium tremens*	–	–
178	1263R	53 years	M	Pulmonary TB	–	–	–	+
179	1264	45 years	M	Pulmonary TB	–	–	–	+
180	1266R	c. 77 years	M	Senility, lues	Pulmonary TB	Syphilis	Parkinson’s disease, arteriosclerosis	–

No.	Terry No.	Age at death	Sex	Cause of death				Exhibiting GIs or not
				MR	DC1	DC2	DC3	
181	1275	c. 59 years	M	Pulmonary TB, myocarditis	Pneumonia	Arteriosclerosis	–	–
182	1278	45 years	M	Pulmonary TB	–	–	–	–
183	1282	61 years	M	Myocarditis	Pulmonary TB	Myocarditis	–	–
184	1285	36 years	M	TB	–	–	–	–
185	1287	24 years	F	Pulmonary TB	–	–	–	–
186	1300	28 years	M	TB	–	–	–	–
187	1304R	58 years	M	Pulmonary TB	Pulmonary TB	Pericardial effusion	–	+
188	1309	36 years	M	Neurological condition	TB spondylitis	Nephritis	Paralysis of the lower extremities	+
189	1313	63 years	M	TB	Syphilis	Pulmonary TB	Myocarditis, arteriosclerosis	–
190	1315	43 years	M	TB	–	–	–	–
191	1318	43 years	M	Pulmonary TB	–	–	–	+
192	1319	c. 36 years	M	Pulmonary TB	Pulmonary TB	Syphilis	–	+
193	1322	34 years	M	Pulmonary TB	–	–	–	–
194	1331	41 years	M	Pulmonary TB	–	–	–	+

No.	Terry No.	Age at death	Sex	Cause of death				Exhibiting GIs or not
				MR	DC1	DC2	DC3	
195	1337RR	35 years	F	Psychosis	Pulmonary TB	Brain syndrome	–	–
196	1346	73 years	M	Lung abscess	Pulmonary TB	–	–	–
197	1352	28 years	M	Pulmonary TB	–	–	–	–
198	1359	52 years	M	Bronchitis, alcoholism	Pulmonary TB	–	–	–
199	1362	36 years	M	Pulmonary TB	–	–	–	–
200	1367	32 years	M	Pulmonary TB	–	–	–	–
201	1369	52 years	M	Pulmonary TB	–	–	–	–
202	1377	c. 52 years	F	Haemorrhage	Haemorrhage into the right ventricle	TB meningitis	–	–
203	1379	c. 54 years	M	Myocarditis	Myocarditis	Pulmonary TB	–	–
204	1388	29 years	M	Pulmonary TB	Pulmonary TB	TB peritonitis	Cardiac decompensation	+
205	1397	35 years	M	TB	–	–	–	–
206	1398	31 years	M	TB, meningitis	–	–	–	–
207	1401	47 years	F	Pulmonary TB	–	–	–	–
208	1406	63 years	F	TB	–	–	–	–

No.	Terry No.	Age at death	Sex	Cause of death				Exhibiting GIs or not
				MR	DC1	DC2	DC3	
209	1407	44 years	M	TB	–	–	–	–
210	1419	54 years	F	Pulmonary TB	–	–	–	–
211	1422R	c. 35 years	M	Pulmonary TB	–	–	–	–
212	1428R	71 years	M	Pulmonary TB	–	–	–	–
213	1434R	19 years	F	Pulmonary TB	–	–	–	–
214	1451	36 years	M	TB	–	–	–	–
215	1453R	58 years	M	Pulmonary TB	–	–	–	–
216	1455	68 years	M	TB	TB	Pneumonia	–	–
217	1458	37 years	M	Pulmonary TB	–	–	–	+
218	1476	51 years	F	Pulmonary TB, TB adenitis	–	–	–	–
219	1503	22 years	M	Pulmonary TB	–	–	–	+
220	1507	23 years	F	Pulmonary TB, heart disease	–	–	–	–
221	1521	54 years	M	Pulmonary TB	–	–	–	–
222	1531	40 years	M	Pulmonary TB	–	–	–	–

No.	Terry No.	Age at death	Sex	Cause of death				Exhibiting GIs or not
				MR	DC1	DC2	DC3	
223	1533	45 years	M	Pulmonary TB	–	–	–	+
224	1536	71 years	F	Pulmonary TB	–	–	–	–
225	1539	23 years	M	Pulmonary TB	–	–	–	–
226	1544	c. 24 years	F	TB	Pulmonary TB	–	–	–
227	1551	25 years	F	?	Salpingitis	TB peritonitis	–	–
228	1553	30 years	F	Possible TB, heart disease	Nephritis	–	–	–
229	1555	41 years	F	Pulmonary TB	–	–	–	–
230	1562	46 years	F	Pulmonary TB	–	–	–	–
231	1568	c. 62 years	F	TB	Pulmonary TB	–	–	–
232	1572	c. 44 years	F	Pulmonary TB	–	–	–	+
233	1576	69 years	F	Pulmonary TB	Pulmonary TB	Schizophrenic reaction	–	–
234	1629	80 years	F	Pneumonia	Pulmonary TB	–	–	–
