# Supplementary material for: Tracking down the White Plague: The skeletal evidence of tuberculous meningitis in the Robert J. Terry Anatomical Skeletal Collection
Source: PLoS One. 2020 Mar 18;15(3):e0230418. doi: 10.1371/journal.pone.0230418 (PMC7080279; doi:10.1371/journal.pone.0230418)
Supplement: S3 Table — Distribution of individuals exhibiting GIs in the Terry Collection by affected cranial bones (considering the left and right greater wings of the sphenoid bone as two separate bones), extent, and number of lesions (L = left, R = right). Number of individuals in the A) TB group and B) NTB group. (PDF) [file pone.0230418.s003.pdf]

S3 Table: Distribution of individuals exhibiting GIs in the Terry Collection by affected cranial bones (considering the left and right greater wings of the sphenoid bone as two separate bones), extent, and number of lesions (L = left, R = right). Number of individuals in the A) TB group and B) NTB group.

A) TB group (Σ=234)		Frontal bone	Parietal bone (L)	Parietal bone (R)	Temporal bone (L)	Temporal bone (R)	Sphenoid bone (L)	Sphenoid bone (R)	Occipital bone
		32/68 (47.06%)	6/68 (8.82%)	10/68 (14.71%)	20/68 (29.41%)	20/68 (29.41%)	10/68 (14.71%)	19/68 (27.94%)	62/68 (91.18%)
Extent (x) of lesions	x < 25%	31/32 (96.88%)	6/6 (100.00%)	10/10 (100.00%)	18/20 (90.00%)	14/20 (70.00%)	9/10 (90.00%)	11/19 (57.89%)	50/62 (80.65%)
	25% ≤ x < 50%	1/32 (3.13%)	–	–	2/20 (10.00%)	2/20 (10.00%)	1/10 (10.00%)	7/19 (36.84%)	11/62 (17.74%)
	50% ≤ x < 75%	–	–	–	–	4/20 (20.00%)	–	1/19 (5.26%)	1/62 (1.61%)
	75% ≤ x	–	–	–	–	–	–	–	–
Number of lesions	Unifocal	8/32 (25.00%)	4/6 (66.67%)	6/10 (60.00%)	9/20 (45.00%)	9/20 (45.00%)	9/10 (90.00%)	14/19 (73.68%)	9/62 (14.52%)
	Multifocal	24/32 (75.00%)	2/6 (33.33%)	4/10 (40.00%)	11/20 (55.00%)	11/20 (55.00%)	1/10 (10.00%)	5/19 (26.32%)	53/62 (85.48%)

B) NTB group (Σ=193)		Frontal bone	Parietal bone (L)	Parietal bone (R)	Temporal bone (L)	Temporal bone (R)	Sphenoid bone (L)	Sphenoid bone (R)	Occipital bone
		4/6 (66.67%)	1/6 (16.67%)	1/6 (16.67%)	2/6 (33.33%)	3/6 (50.00%)	1/6 (16.67%)	1/6 (16.67%)	6/6 (100.00%)
Extent (x) of lesions	x < 25%	4/4 (100.00%)	1/1 (100.00%)	1/1 (100.00%)	2/2 (100.00%)	3/3 (100.00%)	1/1 (100.00%)	1/1 (100.00%)	5/6 (83.33%)
	25% ≤ x < 50%	–	–	–	–	–	–	–	–
	50% ≤ x < 75%	–	–	–	–	–	–	–	1/6 (16.67%)
	75% ≤ x	–	–	–	–	–	–	–	–
Number of lesions	Unifocal	–	–	–	–	–	1/1 (100.00%)	1/1 (100.00%)	1/6 (16.67%)
	Multifocal	4/4 (100.00%)	1/1 (100.00%)	1/1 (100.00%)	2/2 (100.00%)	3/3 (100.00%)	–	–	5/6 (83.33%)
