# Supplementary material for: Tracking down the White Plague: The skeletal evidence of tuberculous meningitis in the Robert J. Terry Anatomical Skeletal Collection
Source: PLoS One. 2020 Mar 18;15(3):e0230418. doi: 10.1371/journal.pone.0230418 (PMC7080279; doi:10.1371/journal.pone.0230418)
Supplement: S4 Table — (APDIs = abnormally pronounced digital impressions; ABVIs = abnormal blood vessel impressions; PAs = periosteal appositions; + = present; − = not present). (PDF) [file pone.0230418.s004.pdf]

S4 Table: Individual data of cases exhibiting GIs regarding other probable TBM-associated endocranial bony changes in the NTB group (Σ=6). (APDIs = abnormally pronounced digital impressions; ABVIs = abnormal blood vessel impressions; PAs = periosteal appositions; + = present; − = not present)

No.	Terry No.	APDIs	ABVIs	PAs
1	4R	−	−	−
2	197R	+	−	−
3	272	+	−	+
4	465	+	−	−
5	506	+	−	−
6	1378	−	−	−
