# Supplementary material for: Tracking down the White Plague: The skeletal evidence of tuberculous meningitis in the Robert J. Terry Anatomical Skeletal Collection
Source: PLoS One. 2020 Mar 18;15(3):e0230418. doi: 10.1371/journal.pone.0230418 (PMC7080279; doi:10.1371/journal.pone.0230418)
Supplement: S5 Table — (PNBFs = periosteal new bone formations; HPO = hypertrophic pulmonary osteopathy; + = present; − = not present) (PDF) [file pone.0230418.s005.pdf]

S5 Table: Individual data of cases exhibiting GIs regarding possible TB-related non-endocranial bony changes in the NTB group (Σ=6).
(PNBFs = periosteal new bone formations; HPO = hypertrophic pulmonary osteopathy; + = present; − = not present)

No.	Terry No.	PNBFs on the visceral costal surfaces	HPO	Extra-spinal osteomyelitis	Extra-spinal arthritis	Vertebral hypervascularization	Vertebral lytic lesions and/or arthritis	Reactive new bone formations indicative of a cold abscess
1	4R	−	−	−	−	−	−	−
2	197R	−	−	−	−	−	−	−
3	272	−	−	−	−	−	−	−
4	465	+	−	+	−	+	−	−
5	506	+	+	+	−	−	−	−
6	1378	−	−	−	−	+	−	−
