# Supplementary material for: Tracking down the White Plague: The skeletal evidence of tuberculous meningitis in the Robert J. Terry Anatomical Skeletal Collection
Source: PLoS One. 2020 Mar 18;15(3):e0230418. doi: 10.1371/journal.pone.0230418 (PMC7080279; doi:10.1371/journal.pone.0230418)

S1 Fig. Demographic profile of individuals surveyed in the Terry Collection.
Number of individuals in the A) TB group (Σ=234) and B) NTB group (Σ=193) by age at death and sex.

A)

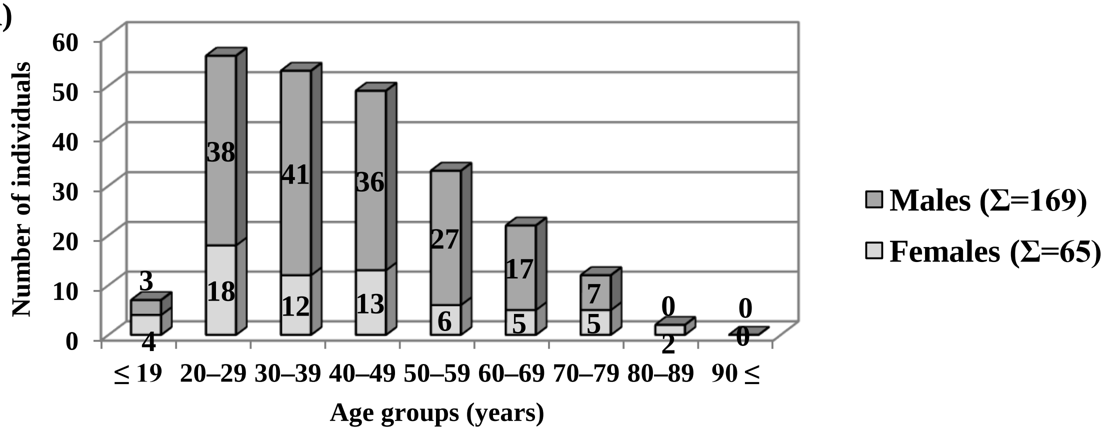

B)

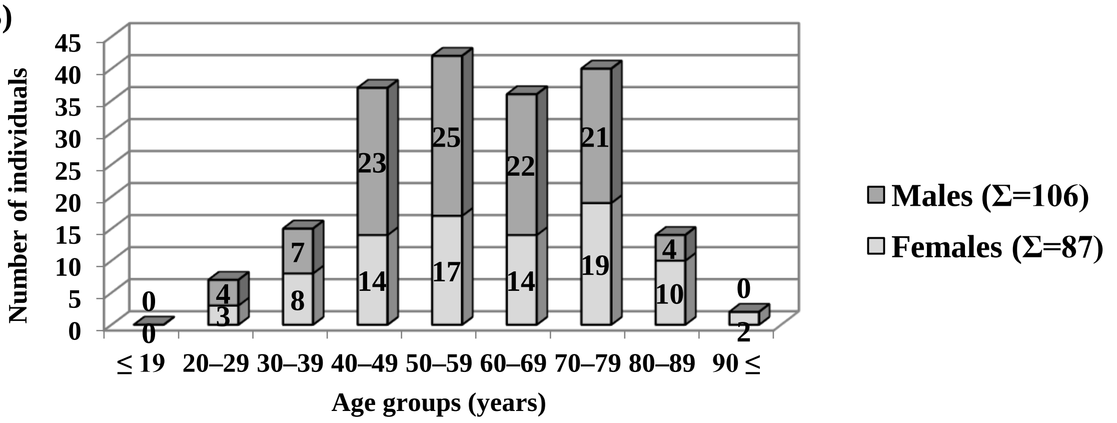

Supplement: S1 Fig — Number of individuals in the A) TB group (Σ = 234) and B) NTB group (Σ = 193) by age at death and sex. (PDF) [file pone.0230418.s006.pdf]
